# Can We Accurately Time the Administration of Antenatal Corticosteroids for Preterm Labor?

**DOI:** 10.1155/2016/5054037

**Published:** 2016-11-07

**Authors:** Paola Aghajanian, Quy T. Nguyen, Naomi H. Greene, Kimberly D. Gregory

**Affiliations:** Division of Maternal-Fetal Medicine, Department of Obstetrics and Gynecology, Cedars-Sinai Medical Center, Los Angeles, CA 90048, USA

## Abstract

*Background*. Accurate timing of antenatal corticosteroids (ACS) has resulted in improved neonatal outcomes.* Objectives*. Our primary objective was to determine predictors for optimal timing of ACS in women presenting with spontaneous preterm labor.* Study Design*. A retrospective cohort study of women receiving ACS for spontaneous preterm birth was conducted. Women were included if they presented with preterm labor or preterm premature rupture of membranes. Accurate timing of ACS was defined as administration within 7 days of delivery. Maternal demographic and obstetrics characteristics were compared between the groups receiving ACS ≤7 days and >7 days from delivery. Statistical analyses were performed using parametric and nonparametric tests. *P* < 0.05 was considered significant.* Results*. The study included 215 subjects. Median latency from ACS administration to delivery was 6 days (IQR 32). Accurate timing of ACS occurred in 113 (53%) women and was associated with rupture of membranes (OR 13.8, 95% CI 5.9–32.6), cervical change (OR 7.1, 95% CI 3.0–17.1), and cervical dilation ≥ 2 cm (OR 3.9, 95% CI 1.5–10.3).* Conclusions*. Rupture of membranes, cervical change, and cervical dilation ≥ 2 cm were strong predictors of optimal timing. 53% of women with preterm labor received ACS optimally.

## 1. Introduction

Antenatal corticosteroids (ACS) are one of the most effective prenatal interventions available for the prevention of perinatal morbidity and mortality related to preterm birth. Since Liggins and Howie first demonstrated the positive neonatal effects of ACS administration in 1972, multiple trials have validated the therapy [1, 2]. ACS significantly reduces the incidence of respiratory distress syndrome (RDS) and neonatal death related to RDS and is associated with a reduction in intraventricular hemorrhage and necrotizing enterocolitis. The beneficial effects of ACS may decrease as the ACS-to-delivery interval exceeds 7 days [3]. If the ACS-to-delivery interval is greater than 14 days, neonates who are delivered at >28 weeks require increased rates of ventilator support and surfactant use [4]. Infants ≤ 32 weeks delivered >7 days following ACS exhibited decreased respiratory compliance compared with those treated within 7 days of delivery [5]. In another study, infants delivered more than 7 days after a single course of ACS demonstrated an increased need for short-term respiratory support [6]. Repeated courses of steroids may improve fetal lung function but at the expense of decreased fetal growth and head size. Following a rescue course, multiple doses of ACS are not recommended [7].

When to administer ACS has been a dilemma for clinicians due to the difficulty in identifying women with preterm labor who will go on to give birth preterm [8]. In fact, 30% of preterm labor resolves spontaneously and half of women who present with preterm labor go on to deliver at term [7]. The American College of Obstetricians and Gynecologists and the NIH Health Consensus Development Panel recommends that a single course of ACS be administered between 24 and 34 weeks of gestation to women at risk of delivery within 7 days [7, 9]. Clinicians therefore encounter the challenge of administering the first course of ACS within 7 days of delivery to achieve the maximum neonatal benefit yet not missing the opportunity to administer it altogether. At this time few studies exist to guide the clinician in appropriately timing the first course of ACS for patients hospitalized with preterm labor. Studying the predictors for accurate timing of ACS in the setting of preterm labor could provide an opportunity to improve the timing of its administration and hence lead to optimization of its effects. Our primary objective was to determine predictors for optimal timing of ACS, defined as delivery >24 hours and ≤7 days after the second dose, in women presenting with spontaneous preterm labor. Our secondary objective was to evaluate the proportion of patients presenting with spontaneous preterm labor or preterm premature rupture of membranes (PPROM) who were given ACS optimally.

## 2. Materials and Methods

We conducted a retrospective cohort study of women who delivered between November 1, 2012, and November 30, 2014, in our tertiary care center. Women were included if they received their first complete ACS course for spontaneous preterm labor at 24 to 34 weeks of gestation. This gestational time period was chosen because at the time the study was conducted, it was the recommended time frame for administration of antenatal corticosteroids for fetal lung maturity. Women with threatened preterm labor or PPROM were included. Patients were excluded if there were medical or fetal indications for delivery because most of these patients had a planned delivery 48 hours or sooner after the first dose of ACS. The maternal and neonatal charts were reviewed for maternal demographics, obstetric characteristics, and neonatal outcomes. Demographic characteristics included age, gravidity, parity, prior preterm birth, singleton or multiple gestation, body mass index, ethnicity, assisted reproductive technology, preexisting diabetes, chronic hypertension, or smoking. Neonatal outcomes included birth weight, head circumference, and NICU admission. Obstetric characteristics noted at the time of admission were reviewed including cervical dilation, rupture of membranes, vaginal bleeding, contractions on tocometer, use of tocolytic agents through the steroid window, and presence of an abnormal fetal heart tracing. Fetal fibronectin results and cervical length were recorded if available. The latency period was calculated from the time of the last dose of the initial course of antenatal corticosteroids to the time of delivery.

For the analysis, we categorized all eligible patients into an optimally timed group with a latency period ≤7 days and a suboptimally timed group with a latency period >7 days. We performed univariate analyses comparing maternal and obstetric characteristics between optimal and suboptimal timing groups using variable-appropriate tests (chi-square, Fisher's exact test). In addition, we performed multivariate logistic regression to assess the predictors for optimal timing of ACS. A two-sided *P* < 0.05 was considered significant. Results were expressed as an odds ratio (OR) with 95% confidence interval (CI), mean ± standard deviation (SD), or median with interquartile range (IQR). All analyses were performed using SAS 9.3 (Cary, NC USA). The study was approved by the Institutional Review Board at Cedars-Sinai Medical Center.

## 3. Results

A total of 409 women delivered at our institution during our study period and received ACS. We excluded 194 patients due to the presence of a medical or fetal indication for delivery. 215 patients who were then categorized into two groups with 113 (53%) being classified into the optimally timed group and 102 (47%) being classified into the suboptimally timed group were included in our analysis. Betamethasone was given to 214 of 215 patients whereas one patient received dexamethasone. Of the 215 patients, 75 patients presented with PPROM. Optimal timing of ACS administration was noted in 57 (76%) of PPROM patients. In the entire cohort, mean maternal age was 33 ± 6 years and median body mass index was 27.1 kg/m^2^ (IQR 6.3). The median gestational age at delivery was 32.0 weeks (IQR 4.9) for the optimally timed group and 36.0 weeks (IQR 4.6) for the suboptimally timed group (*P* < 0.001). The median latency period was 1 day (IQR 2) for the optimally timed group and 35 days (IQR 32) for the suboptimally timed group (*P* < 0.001). There was no difference in the number of patients with a history of prior preterm delivery in the two groups (Table 1). A rescue course of ACS was given to 13 (6%) patients, all in the suboptimally timed group.

Rupture of membranes (*P* < 0.01), cervical dilation ≥ 2 centimeters (*P* < 0.001), cervical change (*P* < 0.001), and presence of regular contractions on the tocometer at the time of admission (*P* = 0.02) were significantly associated with optimal timing of ACS in the univariate analysis. Prior preterm birth, vaginal bleeding, multifetal pregnancy, and use of tocolytics were not associated with optimal ACS administration (Table 2). Multivariate logistic regression was performed in order to control for potential confounders and revealed PPROM to be a strong predictor of optimal ACS timing (OR, 13.8; 95% CI, 5.9–32.6). When analyzing only patients with spontaneous preterm labor and intact membranes, cervical change (OR, 8.0; 95% CI, 3.0–21.7) and cervical dilation ≥ 2 centimeters (OR, 6.1; 95% CI, 2.1–18.1) remained independently associated with optimal timing of ACS (Table 3).

Of all 215 patients, 10 patients demonstrated all 4 of the above significant predictors on admission, 29 patients had 3 of the predictors, 63 patients had 2 of the predictors, 84 patients had 1 predictor, and 29 (13%) patients had none of the four predictors.

## 4. Comment

In our study, among patients presenting with preterm labor with or without intact membranes, 53% received a complete course of ACS optimally, defined as being within 7 days of delivery. In PPROM patients, 76% received ACS optimally. We found PPROM to be a strong predictor of optimal timing of ACS. In women with intact membranes, cervical change and cervical dilation ≥ 2 centimeters remained strong predictors of optimal timing. If ACS were reserved for only patients presenting with threatened preterm labor who had at least one significant predictor of optimal timing, clinicians would miss the opportunity to administer ACS optimally to 13%. Given the predictors of accurate ACS timing that our study identified, it is important to consider that although objective tests are available for diagnosing PPROM and allowing the clinician to administer ACS accordingly, the diagnosis of preterm labor remains a challenge. Cervical exams with determination of cervical change and dilation are examiner dependent. Cervical length and fetal fibronectin are more objective measures but they are not consistently used in the evaluation of preterm labor.

Our rate of optimal ACS administration is comparable to or higher than that reported in previous studies. Among all live births in Nova Scotia, Canada, optimal ACS receipt increased from 10% in 1988 to 23% in 2012 [10]. Among Dutch women with singleton, twin or triplet pregnancies, and preterm labor who completed a first course of ACS, 32% delivered with optimal timing of ACS. In the same cohort, among the subset of women presenting with PPROM, 51% delivered with optimal timing [11]. In another Dutch study, women presenting with suspected preterm labor and PPROM were treated optimally in 32% and 27% of cases, respectively [12]. In a study by Adams et al., 20% of women receiving ACS for threatened spontaneous preterm labor with or without PPROM delivered within 7 days of administration. PPROM and cervical dilation ≥ 2 cm were noted to be strong predictors of optimal timing as were cervical length ≤ 2 cm and positive fetal fibronectin [13]. The study by Adams et al. however differs from our study in that not all of their patients received a full course of ACS. If only patients with a full course of ACS had been included, then only 11% of women in their study would have received ACS optimally.

Our study has a number of strengths. We reviewed all charts and confirmed all cases of spontaneous preterm labor and admission predictors. Our study also encompasses a large diverse population from a combined academic and private institution and may be generalizable to other community and academic hospital populations. Limitations of our study include the retrospective design and data collection from a single institution. Our hospital does not have a standardized protocol for assessment of women presenting with threatened preterm labor and therefore patients were managed at the discretion of the individual providers. Accordingly, not all patients underwent cervical length measurement or fetal fibronectin testing. Additionally, we chose to include singletons and multifetal gestations in our cohort to maximize sample size and power. However, multifetal pregnancy was not a predictor of accurate timing in the univariate (Table 2) or multivariate analyses (data not shown).

For the reasons already stated, accurate timing of ACS administration can have multiple implications for the neonate including minimizing morbidities related to prematurity. Our results can potentially allow clinicians to choose the best candidates for ACS administration among women presenting with preterm labor while decreasing overutilization of ACS in patients not at risk for delivery within 7 days. Additional research is needed on ways to improve the optimal timing of ACS in preterm labor and whether optimal timing translates into improved neonatal outcomes.

## Figures and Tables

**Table 1:** Comparison of baseline maternal characteristics for mothers in the optimal timing and suboptimal timing groups.

Characteristic	Optimal timing (*n* = 113)	Suboptimal timing (*n* = 102)	*P *value
Age (y), mean ± SD	33.2 ± 6.3	32.8 ± 5.8	0.63
Gravidity, median (IQR)	2 (2)	2 (2)	0.5
Parity, median (IQR)	0 (1)	0 (1)	0.63
Singleton	87 (77%)	79 (78%)	0.94
Multifetal gestation	26 (23%)	23 (23%)	
BMI (kg/m^2^)	27.8 (4.4)	28.3 (5.1)	0.42
Ethnicity			
White	50 (50%)	54 (53%)	0.74
Black	19 (17%)	21 (21%)	
Hispanic	23 (20%)	16 (16%)	
Asian/other	14 (12%)	11 (11%)	
ART	21 (19%)	24 (24%)	0.37
Prior to preterm birth	19 (17%)	12 (12%)	0.29
Smoker	2 (2%)	1 (1%)	1.0
GA at ACS (wks), median (IQR)	31.6 (5.0)	31.6 (3.7)	0.62
GA at delivery (wks), median (IQR)	32.0 (4.9)	36.0 (4.6)	<0.001
Latency (d), median (IQR)	1 (2)	35 (32)	<0.001
Diabetes			
None	105 (93%)	90 (88%)	0.24
Pregestational	1 (1%)	0 (0%)	
Gestational diabetes-A1	3 (3%)	11 (11%)	
Gestational diabetes-A2	4 (4%)	1 (1%)	
Chronic hypertension	1 (1%)	2 (2%)	0.61

BMI, body mass index; ART, assisted reproductive technology; ACS, antenatal corticosteroids; GA, gestational age.

**Table 2:** Predictors of optimal timing of antenatal corticosteroids.

Predictor	Optimal timing (*n* = 113)	Suboptimal timing (*n* = 102)	*P* value
Prior preterm birth	19 (17%)	12 (12%)	0.29
Multifetal gestation	26 (23%)	23 (23%)	0.94
ART	21 (19%)	24 (24%)	0.37
Rupture of membranes	57 (50%)	18 (18%)	<0.001
Vaginal bleeding	20 (20%)	14 (14%)	0.25
Cervical dilation			
<2 cm	77 (68%)	89 (88%)	<0.001
≥2 cm	36 (32%)	12 (12%)	
Cervical change	52 (46%)	14 (14%)	<0.001
Contractions on tocometer	86 (76%)	63 (62%)	0.02
Tocolysis	41 (36%)	41 (40%)	0.56
Abnormal fetal heart tracing	13 (12%)	5 (5%)	0.56
IUGR	1 (1%)	0 (0%)	0.36
IUGR w/abnormal Dopplers	4 (4%)	7 (7%)	

ART, assisted reproductive technology; IUGR, intrauterine growth restriction.

**Table 3:** Multivariable analysis of predictors associated with optimal timing of antenatal corticosteroids.

Predictor	Adjusted odds ratio (95% CI)^a^	*P* value
All with preterm labor (*n* = 215)		
Rupture of membranes	13.8 (5.9–32.6)	<0.001
Cervical change	7.1 (3.0–17.1)	<0.001
Cervical dilation ≥ 2 cm	3.9 (1.5–10.3)	0.005
Contractions on tocometer	1.7 (0.8–3.9)	0.31
Preterm labor with intact membranes (*n* = 140)		
Cervical change	8.0 (3.0–21.7)	<0.001
Cervical dilation ≥ 2 cm	6.1 (2.1–18.1)	0.001
Contractions on tocometer	1.5 (0.5–4.9)	0.48

^a^Adjusted for maternal age, GA at first ACS, Gravidity, BMI, HTN, and ethnicity.

## References

[1] Liggins G. C., Howie R. N. (1972). A controlled trial of antepartum glucocorticoid treatment for prevention of the respiratory distress syndrome in premature infants. *Pediatrics*.

[2] Roberts D., Dalziel S. (2006). Antenatal corticosteroids for accelerating fetal lung maturation for women at risk of preterm birth. *The Cochrane Database of Systematic Reviews*.

[3] Wilms F. F., Vis J. Y., Pattinaja D. A. P. M. (2011). Relationship between the time interval from antenatal corticosteroid administration until preterm birth and the occurrence of respiratory morbidity. *American Journal of Obstetrics and Gynecology*.

[4] Ring A. M., Garland J. S., Stafeil B. R., Carr M. H., Peckman G. S., Pircon R. A. (2007). The effect of a prolonged time interval between antenatal corticosteroid administration and delivery on outcomes in preterm neonates: a cohort study. *American Journal of Obstetrics & Gynecology*.

[5] McEvoy C., Schilling D., Spitale P., Peters D., O'Malley J., Durand M. (2008). Decreased respiratory compliance in infants less than or equal to 32 weeks' gestation, delivered more than 7 days after antenatal steroid therapy. *Pediatrics*.

[6] Peaceman A. M., Bajaj K., Kumar P., Grobman W. A. (2005). The interval between a single course of antenatal steroids and delivery and its association with neonatal outcomes. *American Journal of Obstetrics and Gynecology*.

[7] American College of Obstetricians and Gynecologists (2012). Practice bulletin no. 127: management of preterm labor. *Obstetrics & Gynecology*.

[8] Goldenberg R. L., McClure E. M. (2015). Appropriate use of antenatal corticosteroid prophylaxis. *Obstetrics and Gynecology*.

[9] Antenatal Corticosteroids Revisited: Repeat Courses (2001). National Institutes of Health Consensus Development Conference Statement, August 17-18, 2000. National Institutes of Health Consensus Development Panel. *Obstetrics & Gynecology*.

[10] Razaz N., Skoll A., Fahey J., Allen V. M., Joseph K. S. (2015). Trends in optimal, suboptimal, and questionably appropriate receipt of antenatal corticosteroid prophylaxis. *Obstetrics and Gynecology*.

[11] Vis J. Y., Wilms F. F., Kuin R. A. (2011). Time to delivery after the first course of antenatal corticosteroids: a cohort study. *American Journal of Perinatology*.

[12] Boesveld M., Heida K. Y., Oudijk M. A., Brouwers H. A. A., Koenen S. V., Kwee A. (2014). Evaluation of antenatal corticosteroid prescribing patterns among 984 women at risk for preterm delivery. *Journal of Maternal-Fetal and Neonatal Medicine*.

[13] Adams T. M., Kinzler W. L., Chavez M. R., Fazzari M. J., Vintzileos A. M. (2015). Practice patterns in the timing of antenatal corticosteroids for fetal lung maturity. *Journal of Maternal-Fetal and Neonatal Medicine*.

